# Data on recurrent somatic embryogenesis and *in vitro* micropropagation of *Cnidium officinale* Makino

**DOI:** 10.1016/j.dib.2018.07.013

**Published:** 2018-07-11

**Authors:** Muhammad Adil, Dong Il Kang, Byoung Ryong Jeong

**Affiliations:** a Institute of Agriculture and Life Science, Gyeongsang National University, Jinju 52828, Republic of Korea; b Department of Horticulture, Division of Applied Life Science Graduate School (BK 21 Plus Program), Gyeongsang National University, Jinju 52828, Republic of Korea; c Research Institute of Life Science, Gyeongsang National University, Jinju 52828, Republic of Korea

## Abstract

Cnidium officinale Makino, a perennial herb of the family Umbelliferae, is a well-known medicinal plant in oriental medicine with antidiabetic, tumor metastatic, antiplatelet, antimicrobial and insecticidal properties. Hence, *C. officinale* does not produce seed the plant tissue culture is the viable alternative for its propagation. Node explant from *in vitro* grown *C. officinale* Makino was cultured on MS medium supplemented with plant growth regulators (PGRs) like 2,4-Dichlorophenoxyacetic acid (2,4-D) or/and 6-Benzylaminopurine (BA). It was aimed to investigate the optimal concentration and combination of 2,4-D and BA for somatic embryogenesis in node explant of *C. officinale* Makino. The embryogenic callus was induced on node explant after four weeks in MS medium containing 1.5 mg L^−1^ 2,4-D and 0.5 mg L^−1^ BA. The translucent white, embryogenic callus was subcultured on the respective medium and individual well-structured somatic embryos were observed. Heart and cotyledon stage embryos were pictured under a stereomicroscope. The individual somatic embryos (SE) were transferred to MS medium without PGRs (MS0) and 100% germination was observed. Repeated subculturing of the embryogenic callus for five months resulted in recurrent somatic embryogenesis but with a gradual decline in number.

**Specifications Table**

**Table:** 

Subject area	Plant Biology
More specific subject area	Plant tissue culture and propagation
Type of data	text file, and figures
How data was acquired	ZEISS STEMI 2000-C STEREO MICROSCOPE W/ 3.2MP CAMERA
Data format	Raw
Experimental factors	Not applicable
Experimental features	Five replicates per treatment
Data source location	Gyeongsang National University, Jinju, Republic of Korea
Data accessibility	Data is available within this article

**Value of the data**

● The data proves the role of 2,4-D (auxin) in combination with BA (cytokinin) in somatic embryogenesis from the node explant of the *in vitro* grown *C. officinale*.
● The data of recurrent somatic embryogenesis would be of value for effective clonal propagation of *C. officinale* Makino.
● Also, this data will be important for further detail study of somatic embryogenesis in plants.

## 1 Data

**Fig. 1:**
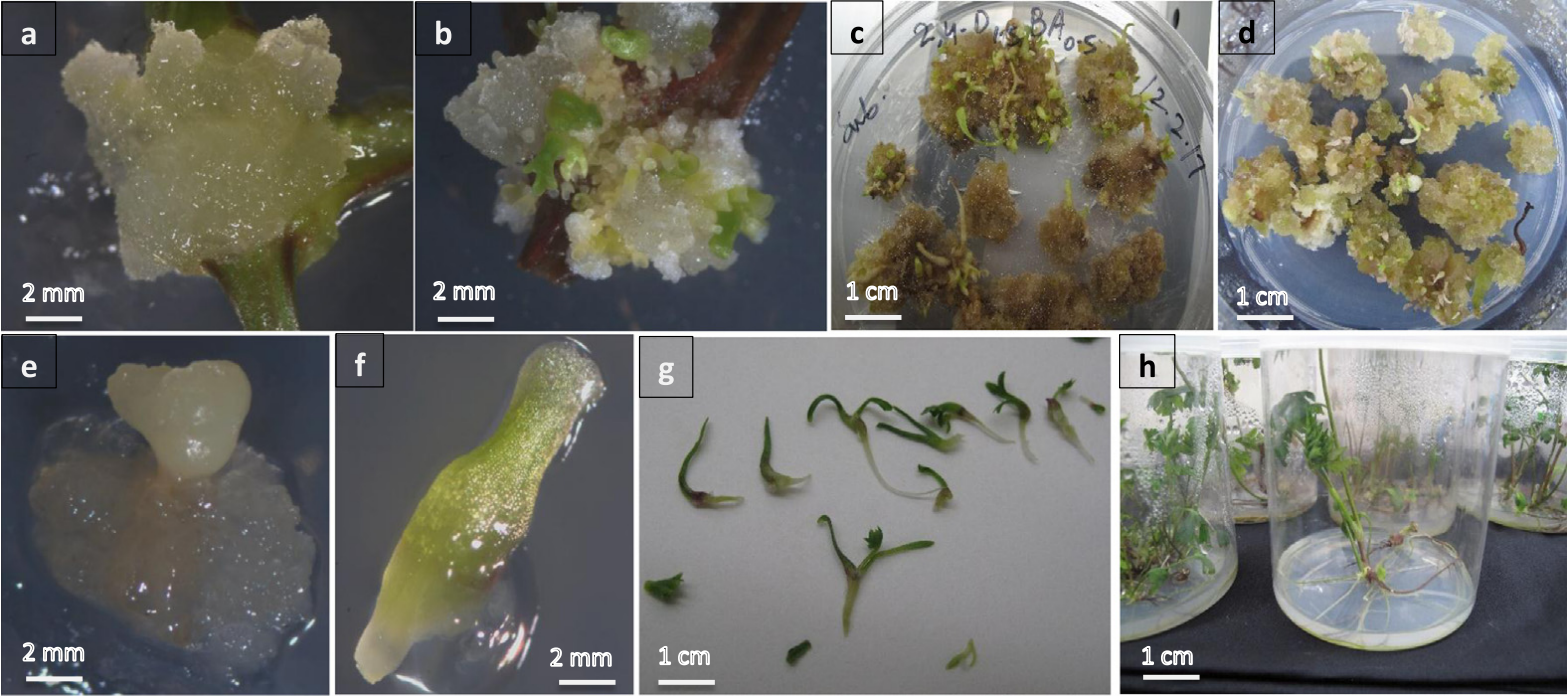
Somatic embryogenesis and micropropagation in *Cnidium officinale* Makino. a Embryogenic translucent callus formation on nodal explant. b Somatic embryos (SEs) formation on obtained callus after 4 weeks of subculture on MS medium containing 2,4-D and BA. c Recurrent somatic embryogenesis on the respective callus after 8 weeks of subculture. d Somatic embryogenesis in callus after 12 weeks of sub-culturing on MS medium containing 2,4-D and BA. e–g Different developmental stages of SEs. h SE-derived plants on PGRs free MS medium.

The nodal explant excised aseptically from *in vitro* grown plant of *Cnidium officinale* Mokino and cultured on MS medium containing 0.5 mg L^−1^ BA and different concentrations of 2,4-D (0.5, 1.0, and 1.5 mg L^−1^). An embryogenic callus was observed after 2-weeks of culture in MS medium containing 0.5 mg L^−1^ BA and 1.5 mg L^−1^ 2,4-D (Fig. 1a). The obtained embryogenic callus was subcultured on respective medium after 4-weeks and somatic embryos (SEs) were observed under the stereomicroscope (Fig. 1b). SE at heart shape and cotyledonary stages were pictured (Fig. 1e and f). Data shows that cultures failed to produce embryos on MS medium containing the lower concentration of 2,4-D (0.5, and 1.0 mg L^−1^) in combination with 0.5 mg L^−1^ BA (Table 2). The individual embryos at the contyledonary stage were transferred to containers filled with 30 mL MS0 medium and 100% conversion to complete plants were detected (Fig. 1g and h). The obtain plants proliferated on MS0 medium in a similar fashion to the mother plant.

**Table 1:** Composition of medium used for somatic embryos (SE) induction and germination.

SE induction media	Ingredients	Amount (g L^−1^)
	MS basal salt (phytotech Ltd.)	4.4
	Sucrose	30
	Agar	8
	Auxin	2,4-D
	Cytokinin	BA
SE germination media	MS basal salt (phytotech Ltd.)	4.4
	Sucrose	30
	Agar	8

**Table 2:** The varying concentrations of auxin (2,4-D) and there combination with cytokinin (BA) effect somatic embryogenesis in *Cnidium officinale* Makino nodal explant.

Treatment (mg L^−1^)		Callus type	Number of SE^a^
2,4-D	BA		
0.5	0.5	Non-embryogenic	–
1.0	0.5	Non-embryogenic	–
1.5	0.5	Embryogenic	>40

a SE: somatic embryos.

## 2 Materials and methods

### 2.1 Plant materials

The *in vitro* grown *C. officinale* Makino was used as the source of nodal explants. These *in vitro* plants were maintained in Horticulture lab at Institute of agriculture science, Gyeongsang National University (GNU), Republic of Korea. The Murashige and Skoog (MS) medium without plant growth regulators (PGRs) was used for *in vitro* plants maintenance in plant growth chamber set at 24 °C (day)/ 18 °C (night) temperature, 16-h photoperiod provided by LED lights and 70% RH [1].

### 2.2 Medium preparation

The Murashige and Skoog (MS) medium was prepared according to the Sharif-Hossain et al. method [2]. Briefly, MS salt was weighed and dissolved in 1000 ml distilled water. Right after mixing, 30 g sucrose was added, followed by PGRs (Table 2) and left for 10 min on a magnetic stirrer. Medium pH was adjusted to 5.7 with 1 N HCl or 1 M NaOH and then finally 8 g tissue culture grade agar was added as a gelling agent. The prepared MS media was sterilized by autoclaving at 121 °C and 15 psi for 20 min [2]. The autoclaved medium containing MS salt, 2,4-D, and BA were termed as induction medium (Table 1). While medium without PGRs was named as germination medium. The sterilized MS medium was poured into petri dishes (90×15 mm^2^) inside laminar flow hood and stocked in dark for future use.

### 2.3 Explant inoculation and somatic embryos induction

*C. officinale* Makino shoot nodes were used as explant for cultures initiation. The excised explant from *in vitro* grown plant was cultured horizontally on the induction medium (Table 1). Each treatment comprised of five petri plates and each plate contained five nodal explants. All cultures were kept in plant growth chamber at 24 °C (day)/18 °C (night) temperature, 16-h photoperiod provided by LED lights and 70% RH. After two weeks of incubation, callus formation was observed that produced somatic embryos upon subculture on the respective media. As a control PGRs free MS medium was used. The cotyledonary stage somatic embryos were isolated and transferred to germination medium. While remaining embryogenic callus masses were subcultured on MS medium containing 1.5 mg L^−1^ 2,4-D and 0.5 mg L^−1^ BA for recurrent somatic embryogenesis. The number of somatic embryos were counted after every 4 weeks of subculture.

### 2.4 Germination of somatic embryos

The isolated well developed cotyledonary SE were transferred into containers containing 50 ml of solid MS medium without PGRs. Five SEs were placed vertically in each container and subcultured onto the fresh MS0 media for another 4 weeks. The percentage of surviving plants were counted.

## References

[1] Murashige T., Skoog F. (1962). A revised medium for rapid growth and bioassays with tobacco tissue cultures. Physiol. Plant.

[2] Hossain A.S., Haq I., Ibrahim N.A., Aleissa M.S. (2016). Callus cell proliferation from broccoli leaf slice using IBA and BAP in vitro culture: its biochemical and antioxidant properties. Data Brief.

