# Supplementary material for: Data on recurrent somatic embryogenesis and in vitro micropropagation of Cnidium officinale Makino
Source: Data Brief. 2018 Jul 11;19:2311–4. doi: 10.1016/j.dib.2018.07.013 (PMC6141485; doi:10.1016/j.dib.2018.07.013)
Supplement: Supplementary file 1 — Supplementary material [file mmc1.docx]

**Conflict of interest**

Dear Editor

We do not have any conflict of interest in publishing the research work in the Data in Brief.

Best Regards

Dr. Byoung Ryong Jeong

Professor, Department of Horticulture,

College of Agriculture & Life Sciences (CALS),

[& Division of Applied Life Science (BK21+ Program), Graduate School]

Gyeongsang National University (GNU)

Jinju, Korea 52828
